# A novel sex-linked mutant affecting tail formation in Hongshan chicken

**DOI:** 10.1038/s41598-017-10943-5

**Published:** 2017-08-30

**Authors:** Qiong Wang, Jinsong Pi, Ailuan Pan, Jie Shen, Lujiang Qu

**Affiliations:** 1 Department of Animal Genetics and Breeding, National Engineering Laboratory for Animal Breeding, College of Animal Science and Technology, China Agricultural University, Beijing, China; 2 Institute of Animal Husbandry and Veterinary Science, Hubei Academy of Agricultural Sciences/Hubei Key Laboratory of Animal Embryonic Engineering and Molecular Breeding, Wuhan, Hubei Province China

## Abstract

The Hongshan chicken is a Chinese indigenous breed that has two distinctly different tail types. Some chickens have stunted tails as compared to the normal phenotype, and they are termed rumpless. Rumplessness in other chicken breeds was caused by a reduction in the number of coccygeal vertebrae. However, X-ray examination showed that rumpless Hongshan chickens possess the normal number of coccygeal vertebrae. Our analyses of the main tail feathers and tissue sections led us to speculate that their stunted tail appearance may be the result of abnormal feather development. To investigate the genetic mechanism underlying rumplessness in Hongshan chickens, we analyzed the results of various crosses. The results indicated that rumplessness is a Z-linked dominant character. In addition, we chose some normal and rumpless individuals for pool-sequencing. Nucleotide diversity and Fst were calculated, and a selective sweep was detected on the Z chromosome. These analyses allowed us to reduce the search area to 71.8–72 Mb on the Z chromosome (galGal5.0). A pseudogene *LOC431648* located in this region appeared a strong candidate involving in Wnt/β-catenin signaling pathway to regulate feather development in chickens.

## Introduction

Beautiful feathers are powerful tools for attracting mates for male birds. Initiation and development of chicken feather provides a useful model for studies on feather growth. Several molecular pathways are involved in feather development^1^. Establishment of feather tracts is the first step in feather formation. Noggin, sonic hedgehog bone morphogenetic protein 2 (BMP2), Wnt, and β-catenin were shown to play a role in this step^2–5^. Thereafter, feather bud formation starts. Wnt-7a, β-catenin, L-fringe, neural cell adhesion molecule (NCAM), Gremlin, and Wnt-11 are involved with a restrictive expression pattern^4–10^. For feather pattern formation, both the activators, fibroblast growth factors (FGF), such as FGF2 and FGF4, and inhibitors of BMPs are necessary^11–13^.

There are several indigenous breeds of chicken with distinct phenotypic traits in China. For instance, Silkies chicken are characterized by dark blue flesh, viscera, and bones and silky feather and Dongxiang blue-shell chicken lays eggs with blue shells. These traits have been investigated in recent years^14, 15^. Indigenous breeds are excellent models for researching the genetic basis of phenotypic diversity, and the sex-linked characters that are useful for studying the Z chromosome evolution or some related issues.

**Figure 1:**
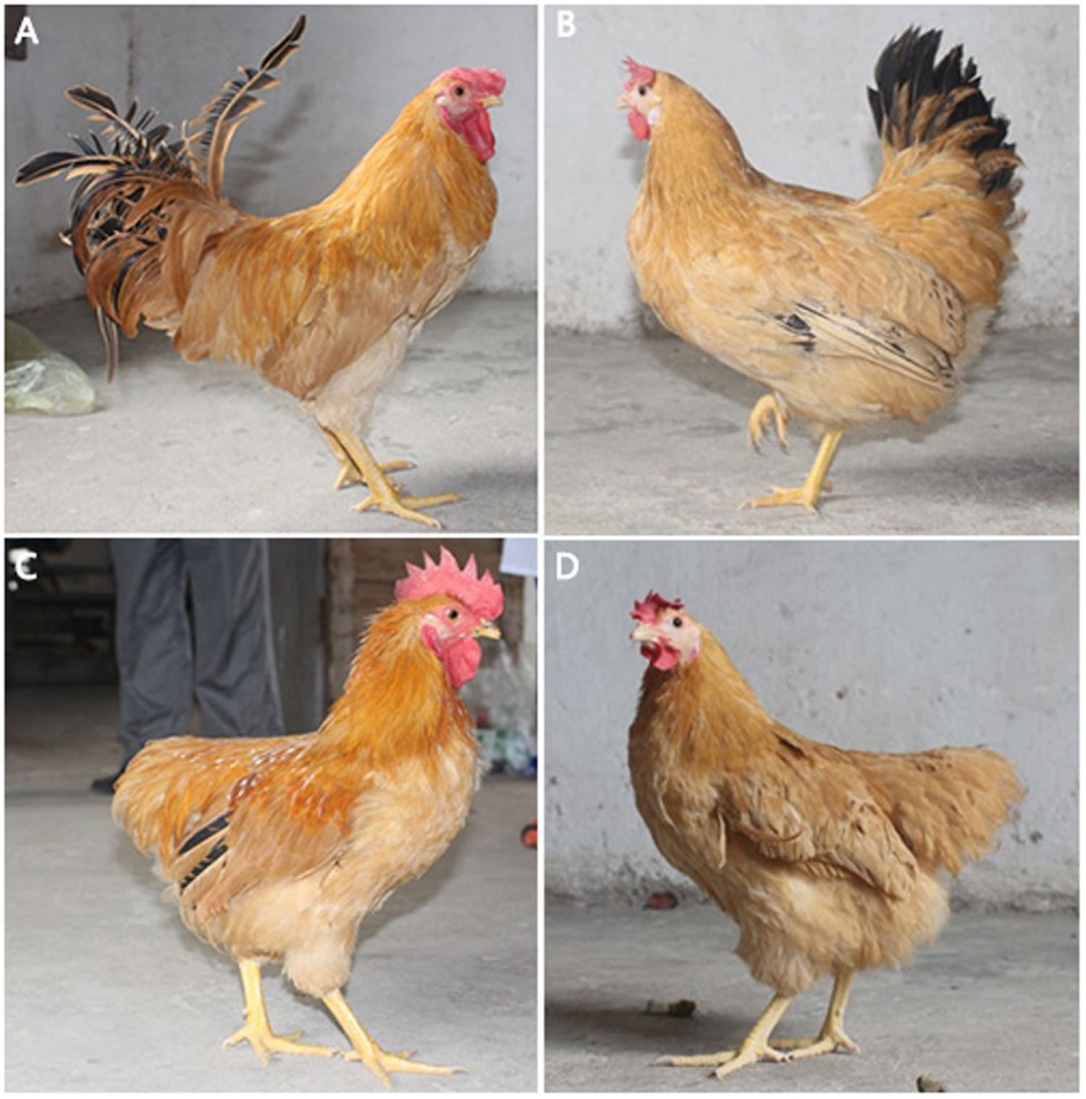
Appearance of the two types of Hongshan chicken. (**A**) Normal male; (**B**) normal female; (**C**) rumpless male; (**D**) rumpless female.

Hongshan chicken is an indigenous dual-purpose breed in Hubei Province, China. The birds are characterized with yellow beaks, shanks, and feathers, but have two distinctly different types of tails^16^. Some chickens have cocked tails, as in other chicken breeds, whereas others have pendulous tails, a condition termed rumplessness (Fig. 1). Roosters with normal tails possess a long sickle feather, and both normal roosters and hens have a greater number of main tail feathers than rumpless chickens have.

Rumplessness phenotypes have been investigated in other chicken breeds and animal species. In some cases, abnormality of coccygeal vertebrae has been identified^17^. A genome-wide association study (GWAS) in Araucana chickens suggested that the rumpless (*Rp*) gene was located on chromosome 2, proximal to the *Iroquois homeobox* genes *IRX1* and *IRX2* ^18, 19^. A recessive type of rumplessness was shown to be controlled by the gene *rp-2*, homozygosity for *rp-2* resulted in a similar phenotype as that of the dominant *Rp* gene^20^. Another type of dominant rumplessness caused by mutation of the *Brachyury* (*T*) gene has been identified^21^ and shown to be characterized by a reduction in the number coccygeal vertebrae. Moreover, mutation of the *T* gene was found to be related to taillessness in mice^22^ and dogs^23^. To the best of our knowledge, the physiological and genetic mechanisms of rumplessness in Hongshan chicken have not been studied. Therefore, the gene or mutation causing the tail malformation in Hongshan chicken is unknown.

Determining the basis of rumplessness in Hongshan chicken may be important for protecting, developing, and utilizing this chicken breed. Therefore, in the present study, we investigated the anatomical changes associated with rumplessness using X-ray imaging and microscopy of tissue sections in order to determine the phenotypic characteristics. Further, we carried out a series of crossing experiments to identify the genetic mechanism of rumplessness. With the rapid development of next-generation sequencing (NGS) technologies over the last few years, whole genome sequencing has become a powerful method for gene mapping^24^. Using NGS, we estimated some population genetic indices to identify the mutation causing rumplessness.

This article has revealed the physiological change in rumpless Hongshan chicken, which was caused by abnormal development of feather. This was a newfound sex-linked mutation in chickens. We provided a suspicious region on Z chromosome by aid of NGS, and a pseudogene in this region probably involving in feather development was reported to be a candidate gene.

## Materials and Methods

### Animals and ethics statement

The birds used in this study were derived from the “Hongshan Chicken Purification and Rejuvenation” breeding base in Hubei Province, where the Hongshan chicken breed were maintained and initiated from 2003. In this breeding base, rumpless and normal chickens were reared and bred separately. The progenies were sequentially classified to two populations after completion of tail development.

The approval for performing the experiments was obtained from the Animal Care and Use Committee of China Agricultural University (Approval ID: XXCB-20090209), and maintenance and housing of the birds conformed to their required standards.

### Body weight

The body weights of Hongshan chickens were measured at different ages. Different groups of chickens were weighed at each age. The frequency of rumplessness was low in our experimental population, therefore, the number of rumpless chickens was lower than that of normal chickens at each age. At the age of 18 weeks, 13 rumpless and 31 normal females were weighed; at 21 weeks, nine rumpless and 30 normal females were weighed; and at 35 weeks, nine rumpless and 21 normal males were weighed.

Two types of Hongshan chicken were raised under same conditions, where an environmentally controlled house with conventional cage system. The temperature was about 20 °C, 2 chickens in each cage, automatic water and free food intake. The diet of each developmental stage was provided by Hubei Tongxing Agricultural Company Limited (Suizhou, China).

### Morphological evaluation

We compared the number of coccygeal vertebrae in normal and rumpless Hongshan chicken using X-ray analysis of 15 rumpless chickens (two females and 13 males) and three normal chickens (two females and one male). Skin samples from around the main tail feather of five rumpless and five normal males were collected and fixed in 4% paraformaldehyde overnight, embedded in paraffin wax, and used to prepare 5-µm sections, which were stained using hematoxylin and eosin (HE), as described previously^25^.

### Inheritance of rumplessness

First, we sought to determine whether rumplessness is a recessive or a dominant trait. We performed four crosses: rumpless × rumpless (cross 1), normal × normal (cross 2), normal × rumpless (cross 3), and rumpless × normal (cross 4); the male parent is listed first.

Moreover, we carried out two crosses using roosters of other breeds and Hongshan rumpless females to confirm the results of the former crosses: sex-linked dwarfism × rumpless (cross 5) and sex-linked green shank × rumpless (cross 6). Both the sex-linked dwarf and green-shanked breeds possess normal tails, and the two traits are Z chromosome linked and are recessive^26–29^.

### Mapping the rumpless allele by whole genomic data

Wing vein blood was obtained from rumpless roosters, normal roosters, and rumpless hens of the same Hongshan population. DNA was isolated from the blood samples using phenol-chloroform protocols. The DNA concentration was determined using a NanoDrop 2000 spectrophotometer (Thermo Fisher Scientific Inc.); three pools (24 rumpless males, 24 normal males, and 27 rumpless female) of DNA were prepared with an equal amount of DNA from each sample. DNA libraries were constructed with approximately 500 bp insert size using TruSeq DNA PCR-Free Sample Prep Kit (Illumina), and 2 × 100 bp paired-end reads were sequenced by the Illumina Hiseq. 2000 protocols (BGI, Shenzhen, China). We obtained abundant clean data. After filtering with NGS QC Toolkit (v2.3)^30^ with default parameters, 191.1 × 10^6^, 187.5 × 10^6^, and 354.9 × 10^6^ high quality read pairs were obtained from rumpless males, normal males, and rumpless females, respectively.

The sequencing data were first mapped to the chicken reference genome (Gallus_gallus-5.0, http://hgdownload.soe.ucsc.edu/downloads.html#chicken) with the Burrows-Wheeler Aligner (BWA)^31^. The BAM files were sorted and duplicate reads removed using Picard toolkit (https://github.com/broadinstitute/picard). The Genome Analysis Toolkit (GAKT)^32^ was used for single-nucleotide polymorphism (SNP) calling.

Three population genetic indices were estimated from the sequence data. First, the nucleotide diversity (π) was used to measure the degree of polymorphism within the population^33^. The second index, fixation index (Fst), measures population differentiation due to genetic structure^34^. Third, selective sweep refers to reduction or elimination of variation among nucleotides near a mutation under strong positive selection^35^. In our analyses, π was calculated by PoPoolation^36^, with parameter set: window size of 10 K, step size of 5 K, minimum allele count of 2, a minimum base quality of 20, a minimum coverage of 10 and a maximum coverage of 200. Fst was calculated by PoPoolation2^37^, with same parameter set as π. SweeD^38^ was used to detect selective sweeps, with default parameters.

### Verification experiments

We collected tail feather follicles from four rumpless and four normal Hongshan chickens three weeks after their tail feathers were removed. Total RNAs were extracted from the tail feather follicles using TRIzol, and reverse transcribed to cDNA using EasyScript One-step gDNA Removal and cDNA Synthesis SuperMix (TransGen Biotech). The expression levels of two genes, *DTWD2* and *LOC431648*, on the Z chromosome were analyzed by quantitative polymerase chain reaction (q-PCR) on an ABI 7500 system (Applied Biosystems, Foster City, CA), with Power SYBR Green PCR Master Mix (Applied Biosystems). All reactions were run in triplicate. Relative gene expression was calculated by the 2^−ΔΔCt^ method^39^. Primer Premier 5^40^ was used to design the q-PCR primers, the product length was set as 100–250 bp. Because *LOC431648* is a pseudogene with high sequence similarity with the mRNA sequence of *OCRL* on chromosome 4, our primers targeted the amplification of the inconsistent regions between *LOC431648* and *OCRL*. We sequenced the amplification products of *LOC431648* to determine whether they were derived from chromosome 4 or Z.

We amplified the whole sequence of the gene *LOC431648*, and extended the sequence to 1500 bp upstream and downstream to search for potential SNPs or structural variations in 12 rumpless and 12 normal Hongshan chickens (six male and six female).

### Data Availability

The datasets generated during and/or analysed during the current study are available from the corresponding author on reasonable request.

## Results

### Body weights of the two types of Hongshan chicken

**Figure 2:**
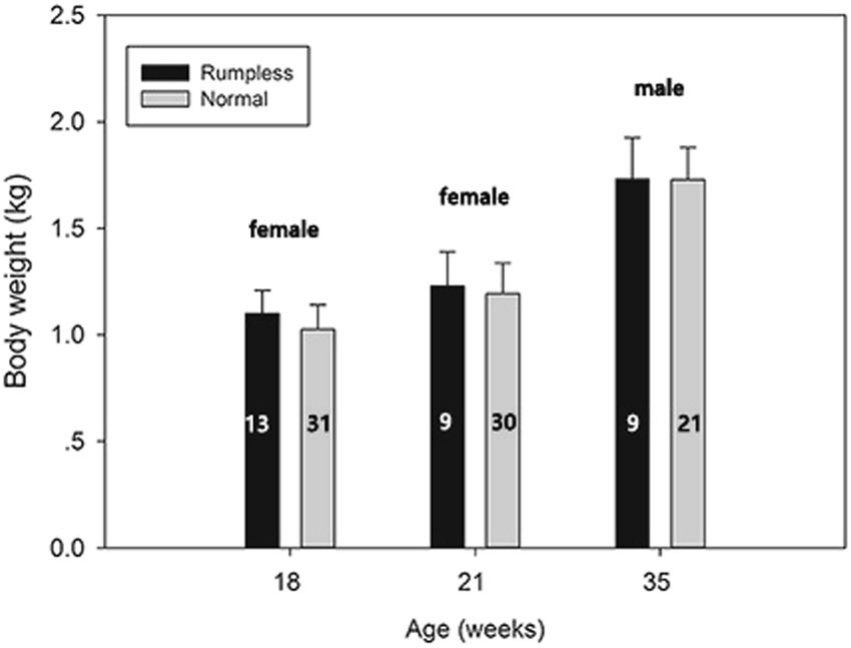
Body weights of the two types of Hongshan chicken at 18, 21, and 35 weeks. The black bars represent rumpless chickens, and the gray bars represent normal chickens. The numbers on the bar indicate sample size. At 18 and 21 weeks, females were measured, and at 35 weeks, males were measured.

No significant differences in body weight were found between rumpless and normal females at 18 or 21 weeks, or in males at 35 weeks (one-way ANOVA) (Fig. 2). At 18, 21, and 35 weeks, the mean body weights of rumpless Hongshan chickens were 1.10, 1.23, and 1.73 kg, respectively, and that of normal Hongshan chicken 1.03, 1.19, and 1.73 kg, respectively.

### Coccygeal vertebral and feather development in rumpless chickens

**Figure 3:**
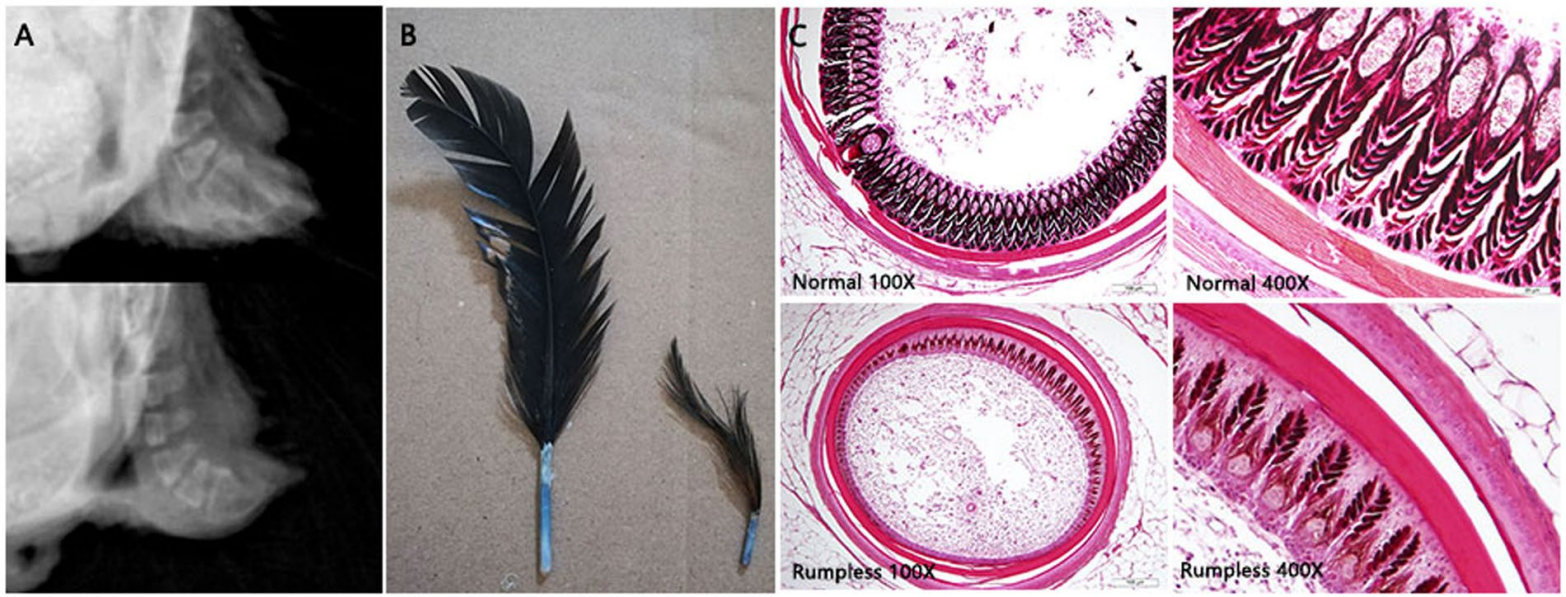
Morphological observations of two types of Hongshan chicken. (**A**) X-ray image of normal (top) and rumpless (bottom) rooster. (**B**) A picture of the main tail feather of normal (left) and rumpless (right) roosters. (**C**) Section of a feather in the feather follicle at 100 × (left) and 400 × magnification (right); normal rooster on top and rumpless rooster at the bottom.

The X-ray analysis showed that rumpless chickens have a normal coccygeal vertebral structure (Fig. 3A), indicating that rumplessness was not caused by variations in skeletal structure. In addition, visual inspection revealed the presence of a normal oil gland on their tails. The altered tail phenotype of rumpless chickens appeared to be the result of changes in the morphology of tail feathers. The main tail feather of rumpless chickens was more slender and frizzy than normal feathers (Fig. 3B,C).

### Rumplessness in Hongshan chicken is a sex-linked dominant character

From the phenotypes of cross 1–4, we found that rumplessness was a Z-linked dominant trait (Table 1). However, there was a lack of male offspring in cross 1–4. Roosters of two other breeds with normal tails in cross 5 and 6 confirmed this result. One of the breeds used in cross 5 and 6 suffered from sex-linked dwarfism, which is a recessive character controlled by the gene *GHR* on the Z chromosome^41^. We obtained 40 offspring, 22 of which were dwarf females with normal tails, 16 rumpless males with normal body size, and the two others were males with normal tail and normal body size. The second breed was a Jianghan chicken that had green shanks, which is a recessive trait determined by an unknown gene on the Z chromosome^14, 42^. We obtained 39 offspring, 17 of which were rumpless and yellow-shanked males, 19 were normal tailed and green-shanked females, two were rumpless and green-shanked males, and one was a normal tailed and yellow-shanked male. Excluding some outliers, our results were similar to the crosses using only Hongshan chickens, which confirmed that rumplessness was a Z-linked dominant trait.

**Table 1:** Progeny phenotypes in six crosses.

Cross	Parents		Son (ZZ)		Daughter (ZW)	
			Norm^1^	Rump^2^	Norm	Rump
1	Male	Rump	1^3^	3	9	9
	Female	Rump				
2	Male	Norm	1	1^3^	21	0
	Female	Norm				
3	Male	Norm	0	5	16	1^3^
	Female	Rump				
4	Male	Rump	2	3	15	15
	Female	Norm				
5	Male	Norm (dwarf)	2^3^	16	22	0
	Female	Rump				
6	Male	Norm (green shank)	1^3^	19	19	0
	Female	Rump				

^1^Normal; ^2^Rumpless; ^3^Outlier, assuming that rumplessness is a Z-linked dominant trait.

### A candidate region on the Z chromosome detected by sequencing data analysis

**Figure 4:**
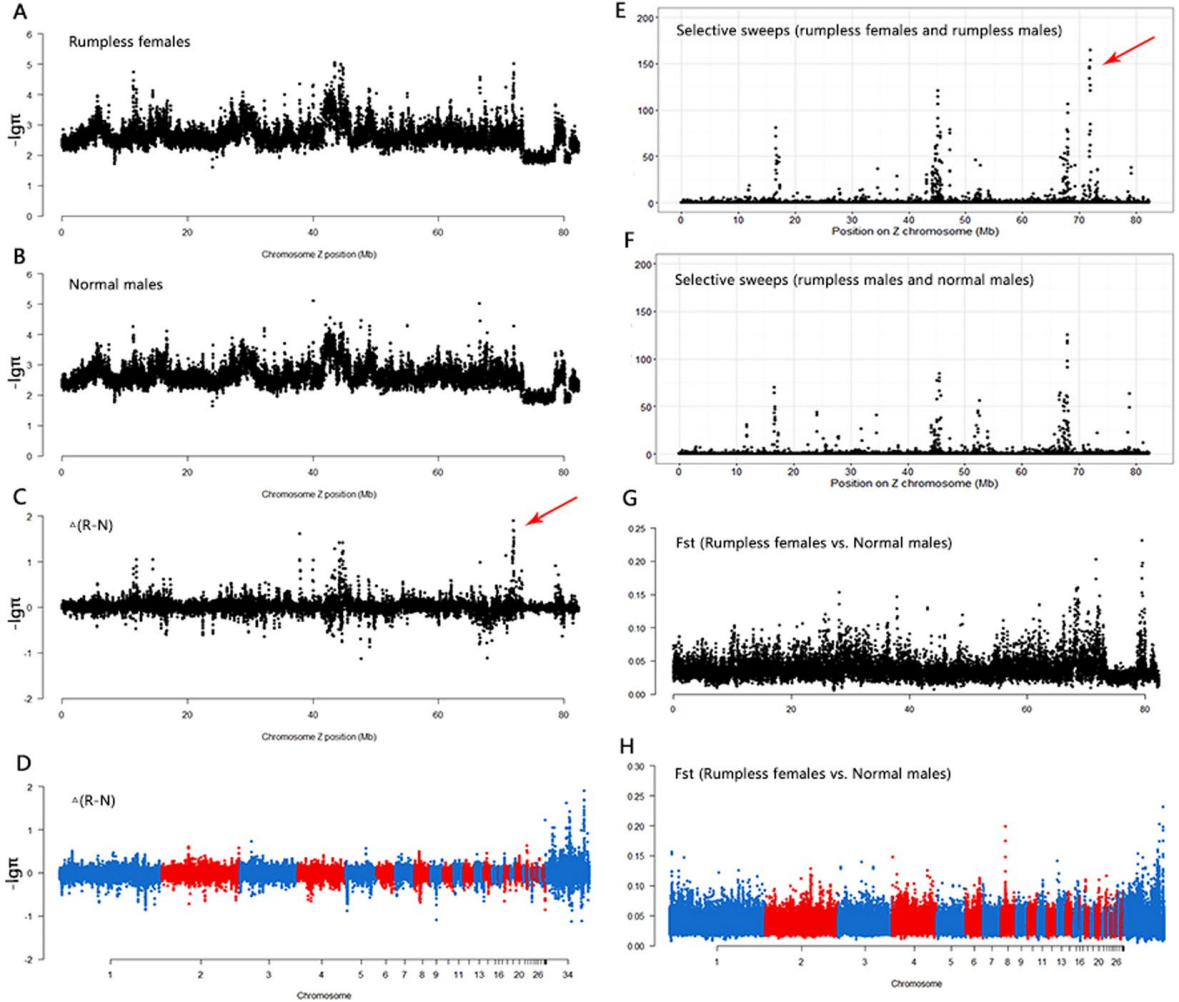
Analysis of three pools of sequencing data (rumpless females, rumpless males, and normal males). Nucleotide diversity on Z chromosome (−log_10_-transformed) of (**A**) rumpless females, and (**B**) normal males. Relative differences between rumpless females and normal males for −log_10_π on (**C**) Z chromosome, and (**D**) genome. Likelihood of selective sweeps on Z chromosome of (**E**) rumpless females and rumpless males, and (**F**) rumpless males and normal males. Fst between rumpless females and normal males on (**G**) Z chromosome and (**H**) genome.

We obtained three pools of sequencing data, namely, rumpless roosters, normal roosters, and rumpless hens. Some population genetic indices were calculated (Fig. 4, Table S1). The π value of 71.8–72 Mb on the Z chromosome in rumpless females was lower than that in normal males; there was a selective sweep at the same region in rumpless chickens. However, the Fst peak was at 79 Mb, and there was a weak signal near 71.8–72 Mb. Two genes were present in this region, DTW domain containing 2 (*DTWD2*; Z, 71760525–71835352) and inositol polyphosphate 5-phosphatase OCRL-1-like (*LOC431648*; Z, 72020597–72022434).

No significant differences were detected between rumpless and normal Hongshan chickens for the expression level of the *DTWD2* and *LOC431648* genes.

Based on sequence variation between *LOC431648* and *OCRL*, our analysis confirmed that the amplification product was derived from *LOC431648*. The complete DNA of *LOC431648* was sequenced, but no fixed SNP was identified within this pseudogene.

## Discussion

Our analyses demonstrate that rumplessness in Hongshan chicken is not caused by changes in coccygeal vertebral structure, and no significant difference was found on body weight, therefore, it is probably not a true example of rumplessness as defined by Dunn (1925). However, as the appearance of this type of Hongshan chicken is similar to rumplessness in other chicken breeds^18^, we shall continue to refer to it as rumpless in the rest of this study.

Because rumplessness in Hongshan chicken is a qualitative trait, we assumed that it was controlled by a single gene. The results of our crossing experiments supported our assumption, and revealed that rumplessness in Hongshan chicken is a Z-linked dominant trait. However, the crosses produced some outliers (Table 1), which might be explained in two possible ways. Firstly, an error in assessment might have led to a normal individual being scored as rumpless because of developmental delay or feather shedding. Secondly, intermediate type existed in the population, most heterozygous individuals were classified as rumpless, but a few of them might be counted as normal ones by mistake.

We sequenced the pools of DNA from rumpless males and normal males at first. Since we found that rumplessness is a sex-linked dominant phenotype, we added a pool of DNA from rumpless females to reduce the effect of heterozygotes in the sequencing analysis, and our analysis focused on the Z chromosome.

The three population genetics indices used in our sequencing data analysis have been proved to be effective in other similar researches. The π has previously been used in analysis of adaptation to high-altitude hypoxia in dogs^43^, and to regulatory mutations that disrupt asymmetric hair pigmentation in horses^44^. Fst has been used to select for sheep without horns (poll)^45^, to analyze head crests in rock pigeon^46^, and to investigate the evolution of vision in chicken^47^. Selective sweep is an efficient method for pool-seq data analysis^48^ and has been used in a study of rabbit domestication^49^.

In our researches, both the π and selective sweep analyses identified a region at 71.8–72 Mb on the Z chromosome, and the Fst analysis identified a closely linked region. Two genes, *DTWD2* and *LOC431648*, close to this region were selected as possible candidates causing rumplessness.

A previous study on genome-wide association showed that several SNPs near the human homolog of *DTWD2* were associated with the maximum number of alcoholic drinks consumed in 24 hours^50^, which may be irrelevant to rumplessness in chickens. Therefore, we focused on the *LOC431648* gene. The *LOC431648* is a processed pseudogene, and its sequence is very similar to the mRNA sequence of *OCRL* on chromosome 4. Although pseudogenes tend to have lost some functionality compared to the intact gene^51^, some are functional and can perform regulatory activities similar to those of noncoding DNA^52^. Our sequencing results showed that *LOC431648* was expressed.

The *OCRL* gene encodes an inositol polyphosphate-5-phosphatase and is involved in inositol phosphate metabolism. Intriguingly, inositol metabolism has a role in the Wnt/β-catenin signaling pathway^53^ that participates in the induction of feather primordia and affects the shape of feather buds in chickens^10^.

We propose that variations in (or near) the *LOC431648* gene cause rumplessness in Hongshan chicken. No differential expression of the gene was detected by q-PCR and no fixed variations in DNA sequence were identified. The feather follicle was collected three weeks after removing the tail feather, when the feather bud had already formed. Therefore, the *LOC431648* expression might have reduced to moderate levels owing to restrictive expression. Besides, the difference in gene expression occurred in a small location of the feather follicle, but the RNA extracted from a mixture of feather follicle and some skin around it might affect the q-PCR results.

Considering that no differential expression of the gene was detected by q-PCR, more powerful tools need to be adopted, like fluorescence *in situ* hybridization (FISH). Furthermore, we cannot exclude the possibility of epigenetic modifications. To investigate these possibilities, further research will be necessary.

## Electronic supplementary material

Table S1
