# Supplementary material for: A novel sex-linked mutant affecting tail formation in Hongshan chicken
Source: Sci Rep. 2017 Aug 30;7:10079. doi: 10.1038/s41598-017-10943-5 (PMC5577132; doi:10.1038/s41598-017-10943-5)
Supplement: Supplementary file 1 — Table S1 [file 41598_2017_10943_MOESM1_ESM.pdf]

A novel sex-linked mutant affecting tail formation in Hongshan chicken

Qiong Wang, Jinsong Pi, Ailuan Pan, Jie Shen, Lujiang Qu

Table S1 The top 50 values of three indexes on Z chromosome

SweeD likelihood top 50 (rumpless females + rumpless males)

Position	Likelihood	Alpha
71848443	203.6353	3.88E-05
71978458	164.2443	2.181E-05
71988459	153.8388	2.41E-05
71828440	146.5956	4.805E-05
71858444	144.7153	3.782E-05
71838441	134.0824	4.321E-05
71968457	126.5797	2.184E-05
45115331	120.5665	1.854E-05
71998460	120.5379	3.277E-05
45105330	114.6007	1.742E-05
68017997	106.4351	1.191E-05
45125332	106.3402	1.826E-05
68027998	97.13919	1.189E-05
68007996	96.65874	1.203E-05
45095329	90.72251	1.913E-05
72018462	84.57109	8.79E-05
45085328	82.32288	2.065E-05
16682022	80.91989	2.745E-05
47285584	78.54339	2.559E-05
67877980	78.38328	2.803E-05
72008461	77.39181	6.25E-05
68037999	76.20554	1.236E-05
45545381	75.59034	2.114E-05
47275583	75.04853	2.543E-05
71818439	73.73971	6.648E-05
45525379	72.05492	2.191E-05
45135333	72.00595	1.903E-05
16692023	71.3694	2.636E-05
45555382	69.71986	2.602E-05
67967991	68.55634	2.104E-05
71958455	61.93937	2.74E-05
45025321	61.67888	1.613E-05
44765290	60.88834	8.437E-06
67867979	60.04707	2.231E-05
67957990	59.06183	2.778E-05
44755289	58.44919	9.296E-06
16672020	58.19291	3.209E-05
47295585	56.64576	2.825E-05
71868445	55.13402	4.597E-05
67977992	54.34726	1.639E-05
67377922	53.46059	3.267E-05
44775292	52.84089	8.497E-06
45015319	52.47038	1.533E-05
68048000	51.45215	1.46E-05
16702024	51.05606	2.757E-05
71768433	49.97295	0.0002074
17262089	49.24325	5.124E-05

66717845	48.82603	8.96E-05
67187900	47.5872	9.656E-05
44745288	47.33035	1.079E-05

Diversity (π) difference top 50 (rumpless females vs. normal males)

Position	snp count-rumpless hens	-lgπ (rumpless hens)	snp count-normal roaster	-lgπ (normal roaster)	Difference
71865000	1	4.5334734	73	2.6269211	1.906552307
71845000	4	4.2730528	81	2.5840883	1.688964593
72005000	2	4.3744818	56	2.6912175	1.683264237
37925000	2	4.3569127	54	2.7397568	1.617155842
72010000	2	4.3685765	41	2.8419329	1.526643657
71960000	1	4.6544694	31	3.1953597	1.459109694
44690000	1	4.8016454	28	3.3835308	1.418114587
44190000	2	4.7745098	29	3.3593069	1.415202867
71995000	1	4.7235612	18	3.3317651	1.391796088
71870000	2	4.1238572	49	2.7830984	1.340758843
43445000	1	5.0529586	5	3.770313	1.282645555
71855000	2	4.1838648	45	2.9302587	1.253606136
44770000	1	4.8537479	14	3.621464	1.232283879
71850000	3	4.1048704	50	2.873716	1.231154447
71820000	4	3.9602251	69	2.7321647	1.22806045
44695000	1	4.4913087	42	3.2828898	1.208418931
71990000	1	4.7304637	12	3.5499773	1.180486369
44705000	1	4.8934352	17	3.7163121	1.177123015
70745000	2	4.2396755	28	3.1019526	1.137722984
71815000	8	3.8717817	65	2.7643842	1.107397527
71840000	16	3.5549924	105	2.4554835	1.099508889
44425000	1	4.9892761	9	3.9234688	1.065807354
71825000	5	3.669359	87	2.611698	1.057660946
14485000	4	4.1340969	35	3.0837256	1.050371304
11845000	4	4.1754642	43	3.1286267	1.046837411
37930000	6	3.8179793	54	2.7720331	1.045946176
40020000	3	4.3141419	15	3.275521	1.038620907
71830000	8	3.6036611	97	2.5653637	1.038297421
44195000	5	4.2389597	41	3.2130875	1.025872168
37920000	9	3.6946744	60	2.6690984	1.025576004
44220000	4	4.2637629	29	3.2489658	1.014797068
44810000	1	4.7046309	14	3.6902272	1.014403631
66560000	1	4.5820801	18	3.5898185	0.992261589
71835000	17	3.4747323	103	2.5015408	0.973191423
44700000	2	4.3507333	35	3.4170576	0.93367574
78600000	11	3.6730766	76	2.7581324	0.914944201
11850000	5	4.0627078	40	3.1615161	0.901191717
44225000	5	4.1691676	27	3.2748425	0.894325124
45135000	7	3.7864344	52	2.9297312	0.856703246
11445000	1	4.7499706	7	3.8975972	0.852373381
44775000	1	4.8689591	6	4.0169965	0.851962556
14480000	4	4.0368026	27	3.1985846	0.838218021
39990000	4	4.2674374	11	3.4382632	0.82917423
43440000	2	4.5639806	6	3.7451354	0.81884511
37935000	16	3.5232596	54	2.7116931	0.811566563

37940000	18	3.4663105	62	2.6580189	0.808291594
73275000	15	3.5397864	67	2.7367011	0.803085363
37945000	18	3.4006858	75	2.6098323	0.790853517
71965000	3	3.9760154	25	3.1887783	0.787237077
11855000	8	3.8074019	51	3.0353346	0.772067327

Fst top 50 (rumpless females vs. normal males)

pos	snp	fst
79465000	45	0.2316531
71615000	60	0.2027129
79470000	32	0.1979087
79450000	70	0.1930344
79445000	48	0.1747455
71610000	91	0.1737044
68550000	47	0.160356
68555000	71	0.1600849
68265000	34	0.1591338
68255000	97	0.1575734
68270000	44	0.1571245
68260000	59	0.1561541
28080000	84	0.1533188
79460000	146	0.1528781
79475000	20	0.1483964
72260000	49	0.1479613
37925000	55	0.1468821
68020000	24	0.143762
68535000	77	0.1426463
68530000	43	0.140207
68760000	61	0.1378412
68310000	92	0.1373799
28075000	104	0.1358336
68540000	80	0.1353712
62035000	41	0.1352686
43015000	4	0.134883
68250000	74	0.1341736
62040000	31	0.1338878
72005000	54	0.132065
68545000	37	0.1309877
67205000	107	0.1304887
43125000	161	0.1304589
37920000	62	0.129014
72000000	31	0.128654
43130000	163	0.128166
68005000	49	0.1281604
79905000	82	0.1279285
68165000	133	0.1271218
72255000	31	0.1262765
68275000	26	0.1260309
79455000	164	0.1249169
67200000	128	0.1247075
70405000	86	0.1242692
72010000	40	0.1237825
68560000	67	0.1237456

79890000	111	0.1225818
72265000	44	0.1216276
70270000	78	0.1213064
68765000	111	0.1203763
25805000	103	0.120166
